# KIF6 p.Trp719Arg Testing to Assess Risk of Coronary Artery Disease and/or Statin Response

**DOI:** 10.1371/currents.RRN1191

**Published:** 2010-11-26

**Authors:** Diane Allingham-Hawkins, Andrew Lea, Susan Levine

**Affiliations:** Hayes, Inc.

## Abstract

Coronary artery disease (CAD) is a leading cause of death worldwide and in the United States it is responsible for more than 500,000 deaths each year. Genome-wide association studies (GWAS) have revealed connections between a number of common single nucleotide polymorphisms (SNPs) and CAD and other cardiovascular diseases. The p.Trp719Arg SNP in the kinesin-like family 6 (KIF6) gene has recently been reported as a potential risk factor for CAD as well as a predictor of response to statin therapy.

## **Clinical Scenario**

Coronary artery disease (CAD), also called coronary heart disease (CHD), is a leading cause of death worldwide, and the most common cause of death in the United States. CAD results from the accumulation of plaque within the walls of coronary arteries, leading to the limitation of blood flow to the heart. Manifestations of CAD include angina, myocardial infarction (MI), heart failure, and arrhythmia. Risk factors associated with cardiovascular disease include age, smoking status, obesity and metabolic syndrome, high low-density lipoprotein cholesterol (LDL-C) or low high-density lipoprotein cholesterol (HDL-C) levels, elevated blood pressure, physical inactivity, insulin resistance, and diabetes mellitus[1]. Genome-wide association studies (GWAS) have identified potential associations between several single nucleotide polymorphisms (SNPs) and risk of CAD[2].The p.Trp719Arg SNP in the kinesin-like family 6 (*KIF6*) gene was first identified as a potential risk factor for CAD in 2007[3] [4]. Subsequent studies have suggested that this SNP may be predictive of response to statin therapy[5] [6] [7]. The potential patient populations for *KIF6* p.Trp719Arg testing include CAD patients with or without a family history of CAD or myocardial infarction (MI), and patients considering statin therapy. 

##  **Test Description**

The *KIF6 *p.Trp719Arg test genotypes the p.Trp719Arg SNP located within the *KIF6* gene. The test is considered positive if the patient is either heterozygous or homozygous for the *KIF6* p.Trp719Arg SNP. The *KIF6 *p.Trp719Arg test is provided by a single laboratory, Berkeley HeartLab Inc.(San Francisco, CA). No specific technical description of the assay method used for *KIF6* genotyping was found on the Berkeley HeartLab website,[8] but several studies describe the general method as allele-specific real-time polymerase chain reaction (PCR)[5] [6] [9] [10]. In the most thorough description found, the technique is characterized as a combination of PCR amplification of target sequences from genomic DNA, followed by allele-specific oligonucleotide ligation. Ligation products are detected using a bead-based microarray system (Luminex Corp.). Using this method, the authors reported an analytical sensitivity of 97.9%[9]. 

## **Public Health Importance**

CAD is the most common cause of death in the United States[1]. In addition, statins are the most commonly prescribed drugs both in individuals with CAD and those at risk for CAD[11]. Consequently, a simple diagnostic test that can effectively predict those at highest risk for CAD and those who are likely to respond favorably to statin therapy has the potential to have a large public health impact. 

## **Published Reviews, Recommendations and Guidelines** 

**Systematic evidence reviews**

None identified.

**Recommendations by independent group**

None identified.

**Guidelines by professional groups**

None identified.

## **Search Strategy**

A literature search of MEDLINE and EMBASE was completed on September 18, 2010, using the search terms (*KIF6 OR kinesin-like family 6 OR kinesin family member 6*) AND (*Trp719Arg* OR *719Arg*) AND (*cardiac OR coronary OR cardiovascular*). After limiting to English language, human, and published since January 1, 1999; this search yielded 7 citations. Citations from relevant references were also reviewed and included as appropriate.

## **Evidence Overview** 

***Analytic Validity*:**Test accuracy and reliability in measuring *KIF6 *p.Trp719Arg genotype. (analytic sensitivity and specificity). 

- No studies that specifically addressed the analytical validity of *KIF6* p.Trp719Arg testing were identified.
- One study claimed an analytical sensitivity of 97.9% using PCR and allele-specific oligonucleotide ligation [9]. However, it is not clear whether this is the methodology used by Berkeley HeartLab as no technical information regarding this test is provided on the laboratory website[8].

***Clinical Validity*:**Test accuracy and reliability in *[supporting clinical or public health assessment]* (predictive value).

- The current body of peer-reviewed research supporting a link between* KIF6* p.Trp719Arg testing and CAD and associated benefit from statin therapy consists of 5 retrospective genetic relationship studies published by authors associated with Celera Corp., the developer of the *KIF6 *p.Trp719Arg test[5] [6] [7] [9] [10].
- The patient populations investigated in these studies consisted primarily of white Americans or Europeans, except for one study that included a relatively small number of nonwhite patients[9]. The overall percentages of *KIF6* p.Trp719Arg carriers (heterozygous and homozygous) across these studies ranged from 50.7% to 62.3%. All of the studies except the Women's Health Study (WHS) investigated the use of statin therapy in at-risk populations.
- Due in part to the variation in endpoints utilized in these studies, it is difficult to draw broad conclusions from the data provided. Reported hazard ratios (HRs) for CAD for *KIF6 *p.Trp719Arg carriers were 1.18 (95% confidence interval [CI] 1.01-1.37; P=0.037) for CHD in the WHS[9], 1.57 (95% CI 1.10-2.25; P=0.01) for MI in the first analysis of the Cholesterol and Recurrent Events (CARE) study[5], and 1.27 (95% CI 0.97-1.66; P=0.08) for MI in patients with prior vascular disease and 0.83 (95% CI 0.60-1.14; P=0.25) in patients without prior vascular disease in the Prospective Study of Pravastatin in the Elderly at Risk (PROSPER) trial[6]. In the West of Scotland Prevention Study (WOSCOPS) study, an odds ratio (OR) of 1.59 (95% CI 1.18-2.14; P=0.003) for a composite cardiovascular endpoint was reported for *KIF6* p.Trp719Arg carriers[5]. These statistics are unadjusted, but significance did not change when adjusted to consider various risk factors such as age, sex, ethnicity, lifestyle factors (e.g. smoking, weight) and cholesterol levels.

- With regards to statin therapy, all studies found that* KIF6* p.Trp719Arg carriers derive significantly more benefit from statin therapy while no significant improvement was reported in noncarriers in any study. In the first analysis of CARE, an absolute risk reduction (ARR) of 4.89% (95% CI 1.81%-7.97%;P=0.002) with pravastatin therapy was reported for *KIF6* p.Trp719Arg carriers, versus an ARR of 1.39% (95% CI –1.94%-4.72%; P=0.41) for noncarriers. In WOSCOPS, ARR for *KIF6* p.Trp719Arg carriers was 5.49% (95% CI 3.52%-7.46%; P<0.0001), versus 0.09% (95% CI –1.97%-2.14%; P=0.99) for noncarriers[5]. In the Pravastatin or Atorvastatin Evaluation and Infection Therapy: Thrombolysis in Myocardial Infarction 22 (PROVE-IT TIMI 22) study, results from intensive statin therapy (atorvastatin 80 milligrams [mg]/day) were compared to moderate statin therapy (pravastatin 40 mg/day). The benefit from intensive therapy was significantly greater in *KIF6* p.Trp719Arg carriers (HR, 0.59; 95% CI 0.45-0.78; P<0.001) versus noncarriers (HR, 0.98; 95% CI 0.72-1.31; P=0.87)[10]. In the PROSPER study, statin therapy reduced cardiovascular events in *KIF6* p.Trp719Arg carriers with prior vascular disease (HR, 0.67; 95% CI 0.52-0.86;P=0.002), but not in noncarriers with prior vascular disease (HR, 0.93; 95% CI 0.68-1.26; P=0.64)[6]. These statistics are unadjusted, but significance did not change when adjusted to consider various risk factors. In a second, more inclusive analysis of the CARE data, it was also found that statin therapy reduced cardiovascular events in *KIF6 *p.Trp719Arg carriers (HR, 0.63; 95% CI 0.49-0.83;P=0.0009), but not in noncarriers (HR, 1.01; 95% CI 0.69-1.45; P=0.98) (only adjusted data presented)[7].  
- In a letter to the editor of the *Journal of the American College of Cardiology*, Stewart and colleagues (2009) presented data that were contradictory to some of the findings published in association with Celera  Corp. The authors analyzed data from the Ottawa Heart Genomics Study (OHGS) and the Wellcome Trust Case Control Consortium (WTCCC) using the more established 9p21.3 variant as a positive control for association with MI risk/CAD, and found no association between the *KIF6* p.Trp719Arg allele and CAD. They also analyzed results from the WHS, CARE, and WOSCOPS studies and felt that the data may support an association between the *KIF6 *p.Trp719Arg allele and MI, but not CAD. These authors did not investigate the association between the *KIF6* p.Trp719Arg allele and benefit from statin therapy[12].

***Clinical Utility*: **Net benefit of test in improving health outcomes.

- No studies that investigated the impact of *KIF6* p.Trp719Arg testing on health outcomes were identified.
- Two ongoing clinical studies related to* KIF6* p.Trp719Arg testing were identified:

- Additional *KIF6* Risk Offers Better Adherence to Statins (AKROBATS) (NCT01068834)[13]: This is a prospective cohort study to determine whether provision of *KIF6* carrier status to study patients will improve adherence to statin therapy. The study is sponsored by Medco Health Solutions Inc. in collaboration with Celera Corp., and is scheduled to be completed in June 2011.
- Genetic Risk Stratification to Identify Individuals for Early Statin Therapy (1RC1HL099634-01)[14]: This ongoing study based at Brigham and Women’s Hospital in Boston, MA, is investigating the use of genetic factors in identifying individuals who would most benefit from statin therapy. The project began in September 2009, and is scheduled to be completed in August 2011.

## **Limitations**

- Imprecision - despite statistically significant results, confidence intervals are wide, suggesting imprecision.
- Patient populations - in most studies, individuals who are homozygous for the p.Trp719Arg variants are combined with heterozygous individuals to create the "carrier" population for analysis. While this is likely to increase the power of the comparisons, it may not be appropriate given that the inheritance pattern has not been clearly established. Indeed, in all of the association studies, homozygotes were not statistically different from controls [5] [6] [9] and in several cases, heterozygotes were not statistically different[6] [9] and it was only when the two populations were combined that significant differences were obtained.
- Conflict of interest - most of the published studies, and all that show a significant association between KIF6 p.Trp719Arg and CAD and/or statin response, were authored by individuals associated with Celera Corp., the parent company of Berkeley HeartLab, thereby creating the potential for conflict of interest.

## **Conclusion**

The current body of evidence shows imprecise, and in some cases, conflicting results. In addition, there is lack of clarity regarding the appropriate patient populationsand the inheritance pattern of the proposed association between *KIF6* p.Trp719Arg and CAD and/or statin response. While the results with respect to statin therapy appear consistent between studies, all studies are retrospective in nature which limits the applicability of the results. Finally, the lack of any studies specifically addressing the clinical utility of this information on patient outcomes is a serious deficiency in the body of evidence. Comparative studies that specifically use genotype information to stratify patients into treatment streams are needed and ideally these studies would be prospective in nature and have sufficient follow-up to determine that outcomes are indeed different in different genotype groups. Therefore, there is currently insufficient evidence to determine the utility of routine testing in patient care. 

****

## **Links**

- **ClinicalTrials.gov:** KIF6
- **RePORTER (Research Portfolio Online Reporting Tool Expenditures and Results)** **:** KIF6

Last updated: October 19, 2010 

## **Acknowledgments**

The authors would like to acknowledge the contributions of the Hayes Genetic Test Evaluation team, particularly Lisa Spock, Linnie Wieselquist and Charlotte Kuo-Benitez. 

## **Funding information**

Funding for the Health Technology Assessment that informed this work was provided by Hayes, Incorporated. Funding to create this Knol was provided by the Centers for Disease Control and Prevention under Contract No. 200-2009-F-32675.This funding was provided through the Genetic Alliance.   

## **Competing interests**

The authors are employees at Hayes, Inc., an independent health technology research and consulting company. None of the employees at this company has any financial or personal interest in any of the technologies reviewed by Hayes, Inc.. No input on report content or conclusions is permitted by manufacturers. Although the CDC funded the work to produce this article, the content is based entirely on Hayes, Inc.'s own analysis and there was no input from the CDC.

## References

[ref-1027605892] U.S. Department of Health & Human Services (DHHS). National Heart, Lung and Blood Institute. 2010. Available at: http://www.nhlbi.nih.gov/. Accessed September 18, 2010

[ref-2122351577] Musunuru K, Kathiresan S. Genetics of coronary artery disease. Annu Rev Genomics Hum Genet. 2010 Sep 22;11:91-108. 20590428 10.1146/annurev-genom-082509-141637

[ref-4247030778] Morrison AC, Bare LA, Chambless LE, Ellis SG, Malloy M, Kane JP, Pankow JS, Devlin JJ, Willerson JT, Boerwinkle E. Prediction of coronary heart disease risk using a genetic risk score: the Atherosclerosis Risk in Communities Study. Am J Epidemiol. 2007 Jul 1;166(1):28-35. Epub 2007 Apr 18. 17443022 10.1093/aje/kwm060

[ref-1325806669] Bare LA, Morrison AC, Rowland CM, Shiffman D, Luke MM, Iakoubova OA, Kane JP, Malloy MJ, Ellis SG, Pankow JS, Willerson JT, Devlin JJ, Boerwinkle E. Five common gene variants identify elevated genetic risk for coronary heart disease. Genet Med. 2007 Oct;9(10):682-9. 18073581 10.1097/gim.0b013e318156fb62

[ref-3191206463] Iakoubova OA, Tong CH, Rowland CM, Kirchgessner TG, Young BA, Arellano AR, Shiffman D, Sabatine MS, Campos H, Packard CJ, Pfeffer MA, White TJ, Braunwald E, Shepherd J, Devlin JJ, Sacks FM. Association of the Trp719Arg polymorphism in kinesin-like protein 6 with myocardial infarction and coronary heart disease in 2 prospective trials: the CARE and WOSCOPS trials. J Am Coll Cardiol. 2008 Jan 29;51(4):435-43. 18222353 10.1016/j.jacc.2007.05.057

[ref-2417930919] Iakoubova OA, Robertson M, Tong CH, Rowland CM, Catanese JJ, Blauw GJ, Jukema JW, Murphy MB, Devlin JJ, Ford I, Shepherd J. KIF6 Trp719Arg polymorphism and the effect of statin therapy in elderly patients: results from the PROSPER study. Eur J Cardiovasc Prev Rehabil. 2010 Aug;17(4):455-61. 20215968 10.1097/HJR.0b013e328336a0dd

[ref-2750138392] Shiffman D, Sabatine MS, Louie JZ, Kirchgessner TG, Iakoubova OA, Campos H, Devlin JJ, Sacks FM. Effect of pravastatin therapy on coronary events in carriers of the KIF6 719Arg allele from the cholesterol and recurrent events trial. Am J Cardiol. 2010 May 1;105(9):1300-5. Epub 2010 Mar 20. 20403483 10.1016/j.amjcard.2009.12.049

[ref-2324953650] Berkeley HeartLab Inc. KIF6. 2009. Available at: http://www.bhlinc.com/clin_test_kif6.php. Accessed September 18, 2010.

[ref-2484280993] Shiffman D, Chasman DI, Zee RY, Iakoubova OA, Louie JZ, Devlin JJ, Ridker PM. A kinesin family member 6 variant is associated with coronary heart disease in the Women's Health Study. J Am Coll Cardiol. 2008 Jan 29;51(4):444-8. Erratum in: J Am Coll Cardiol. 2008 Mar 4;51(9):977. 18222354 10.1016/j.jacc.2007.09.044

[ref-2698703997] Iakoubova OA, Sabatine MS, Rowland CM, Tong CH, Catanese JJ, Ranade K, Simonsen KL, Kirchgessner TG, Cannon CP, Devlin JJ, Braunwald E. Polymorphism in KIF6 gene and benefit from statins after acute coronary syndromes: results from the PROVE IT-TIMI 22 study. J Am Coll Cardiol. 2008 Jan 29;51(4):449-55. 18222355 10.1016/j.jacc.2007.10.017

[ref-1744065251] Ray KK, Seshasai SR, Erqou S, Sever P, Jukema JW, Ford I, Sattar N. Statins and all-cause mortality in high-risk primary prevention: a meta-analysis of 11 randomized controlled trials involving 65,229 participants. Arch Intern Med. 2010 Jun 28;170(12):1024-31. Review. 20585067 10.1001/archinternmed.2010.182

[ref-2603046121] Stewart AF, Dandona S, Chen L, Assogba O, Belanger M, Ewart G, LaRose R, Doelle H, Williams K, Wells GA, McPherson R, Roberts R. Kinesin family member 6 variant Trp719Arg does not associate with angiographically defined coronary artery disease in the Ottawa Heart Genomics Study. J Am Coll Cardiol. 2009 Apr 21;53(16):1471-2. 19371834 10.1016/j.jacc.2008.12.051

[ref-4267717276] Medco Health Solutions Inc. Additional KIF6 Risk Offers Better Adherence to Statins. National Library of Medicine (NLM) Identifier: NCT01068834. Updated February 12, 2010. Available at: http://www.clinicaltrials.gov/ct2/show/NCT01068834. Accessed September 18, 2010.

[ref-3991658313] Brigham and Women's Hospital. Genetic Risk Stratification to Identify Individuals for Early Statin Therapy. Project No. 1RC1HL099634-01. [Search Project Code: 1RC1HL099634%]. RePORTER (Research Portfolio Online Reporting Tool Expenditures and Results) [online database]. 2010. Available at: http://projectreporter.nih.gov/reporter.cfm. Accessed September 18, 2010.

